# Supplementary material for: Hyaluronan-Arginine Interactions—An Ultrasound and ITC Study
Source: Polymers (Basel). 2020 Sep 12;12(9):2069. doi: 10.3390/polym12092069 (PMC7570013; doi:10.3390/polym12092069)
Supplement: Supplementary file 1 [file polymers-12-02069-s001.pdf]

polymers

Supplementary Materials of Hyaluronan-Arginine Interactions—An Ultrasound and ITC Study

Adam Jugl and Miloslav Pekař *

Table S1. A listing of the hyaluronan used for experiments.

Type Name	Batch No.	Humidity	Mw (*) kDa
8–15	213–6147	11%	9
15–30	213–6481	9%	16
80–130	260315-E2	10%	109
130–300	213–6213	10%	137
250–450	120218-E1	5%	310
600–800	210615-A-D2	6%	680
1400–1600	181214–4-D1	9%	1540

M_w (*) is molecular weight obtained by HPLC/SEC-MALS; polydispersity is lower than 1.1, analysis is performed by manufacturer.

Table S2. A listing of monomeric and oligomeric forms of arginine.

Type of Arginine	Properties
Poly-L-arginine hydrochloride (30 Arg·HCl)	Alamanda Polymers (Huntsville, AL, USA), M_w 5.8 kDa, CAS: 26982-20-7, purity: 90-100%, Batch No. 000-R030-103
Poly-L-arginine hydrochloride (12 Arg·HCl)	Chempeptide Limited (Shanghai, China), M_w 2.329 kDa, purity: >95%, Batch No. PT209181809
Poly-L-arginine hydrochloride (10 Arg·HCl)	Chempeptide Limited (Shanghai, China), M_w 1.944 kDa, purity: >95%, Batch No. PT303261912R1
Poly-L-arginine hydrochloride (2 Arg·HCl)	Vidia s.r.o. (Vestec, Czech Republic), M_w 403.31 Da, purity: >95% Batch No. 171103
Poly-L-arginine hydrochloride (4 Arg·HCl)	Vidia s.r.o. (Vestec, Czech Republic), M_w 788.60 Da, purity: >95%, Batch No. 171102
Poly-L-arginine hydrochloride (8 Arg·HCl)	Vidia s.r.o. (Vestec, Czech Republic), M_w 1559.18 Da, purity: >95%, Batch No. 171101
L-arginine monohydrochloride (Arg·HCl)	Sigma Aldrich (St. Louis, MO, USA), 98% (HPLC), CAS: 1119-34-2, Batch No. SLBQ6919V

Table S3. pH of pure arginine oligomer solutions and pH of solutions during titrations at molar ratios of 0.96 and 1.92.

Oligomer	pH of Stock Solution	pH at Molar Ratio 0.96 During Titration	pH at Molar Ratio 1.92 During Titration
2 Arg·HCl	2.00 ± 0.03	3.09 ± 0.05	2.59 ± 0.06
4 Arg·HCl	1.95 ± 0.04	2.94 ± 0.04	2.52 ± 0.06
8 Arg·HCl	1.96 ± 0.02	2.92 ± 0.06	2.56 ± 0.07
Hyaluronan	6.20 ± 0.15		

Table S4. pH adjustment of arginine oligomer solutions.

Oligomer	Initial Volume (mL)	Added Volume of NaOH (µL)	pH after Addition	Concentration of NaCl (mM)
2 Arg·HCl	4.0	70	6.46	34.40
4 Arg·HCl	4.0	60	6.84	29.55
8 Arg·HCl	3.5	50	6.82	28.17

Table S5. Summary of results of individual titrations in water–the visual observation of individual systems.

Arginine Oligomer	Molecular Weight of Hyaluronan / Visual Observation
Dimer and tetramer	unchanged
Octamer	9 kDa–formation of a slightly cloudy solution, which dissolved under higher molar ratios<br>1540 kDa–formation of a slightly cloudy solution, which turned into a precipitate with slight turbidity around a molar ratio of 1.0, it did not fully disintegrate
Octamer pH adjusted	9 kDa–intense turbidity, did not dissolve as in the case of untreated pH<br>1540 kDa–not tested, high-molecular weight hyaluronan was known to interact without pH adjustment
Decamer	9 kDa–formation of a slightly cloudy solution<br>310 kDa–formation of an intensely cloudy solution<br>1540 kDa–formation of a cloudy solution, which turned into a precipitate around the molar ratio of 1.2 and the solution remained clear
Dodecamer	9 kDa–formation of a slightly cloudy solution<br>1540 kDa–the formation of a cloudy solution, which turned into a precipitate around the molar ratio of 1.1 and the solution remained clear
Triacontamer	9 kDa–formation of an intensely cloudy solution, which turned into a precipitate with slight turbidity around the molar ratio of 1.0<br>1540 kDa–formation of an intensely cloudy solution, which turned into a precipitate around the molar ratio of 1 and the solution remained clear

Table S6. Summary of results of individual titrations in PBS or in different NaCl solutions – visual observation of individual systems.

Arginine Oligomer	Molecular Weight of Hyaluronan / Visual Observation
	**PBS**
Triacontamer	9 kDa–formation of a slightly cloudy solution<br>1540 kDa–formation of a slightly cloudy solution, which turned into a precipitate around a molar ratio of 1.5 and the solution remained clear
Dodecamer	The solution remained clear with all the hyaluronan molecular weights tested
Decamer	The solution remained clear with all the hyaluronan molecular weights tested
	**NaCl solution**
Dodecamer 680 kDa hyaluronan	(Water–formation of a cloudy solution, which turned into a precipitate around a molar ratio of 1.1 and the supernatant remained clear)<br>100 mM NaCl–formation of a slightly cloudy solution, which turned into a precipitate with slight turbidity around a molar ratio of 1.1<br>150 mM NaCl–very slight turbidity observable from a molar ratio of 1.5
Decamer 50 mM NaCl	9 kDa–formation of a slightly cloudy solution<br>1540 kDa–formation of an intensely cloudy solution, which turned into a precipitate around a molar ratio of 1 and the supernatantremained clear
Decamer 10 mM NaCl	9 kDa–formation of an intensely cloudy solution<br>1540 kDa–formation of an intensely cloudy solution, which turned into a precipitate around the molar ratio of 1 and the supernatant remained clear

Table S7. Parameters for arginine triacontamer in PBS determined from isothermal titration calorimetry (ITC) (25 °C). N is the molar ratio at the ITC record inflex point (interaction saturation point in the main text); ΔH^o, ΔG^o, and $-T\Delta S^o$ are defined per mole of hyaluronan basic unit.

Oligomer	Hyaluronan Molecular Weight (kDa)	N (-)	ΔH^o (kJ/mol)	K_D (M)	ΔG^o (kJ/mol)	$-T\Delta S^o$ (kJ/mol)
Triacontamer	9	0.93 ± 0.03	0.27 ± 0.03	$(1.1 \pm 0.3)\cdot 10^{-3}$	−17.1 ± 0.6	−17.5 ± 0.6
	16	1.07 ± 0.04	0.37 ±0.05	$(2.4 \pm 0.4)\cdot 10^{-4}$	−20.8 ± 0.4	−21.1 ± 0.4
	109	1.42 ± 0.06	0.44 ± 0.02	$(1.76 \pm 0.14)\cdot 10^{-5}$	−27.2 ± 0.2	−27.6 ± 0.2
	310	1.37 ± 0.04	0.47 ± 0.05	$(3 \pm 5)\cdot 10^{-6}$	−34 ± 4	−34.7 ± 4.3
	680	1.46 ± 0.05	0.46 ± 0.01	$(1.0 \pm 0.0)\cdot 10^{-12}$	−69 ± 0	−69 ± 0
	1540	1.47 ± 0.06	0.49 ±0.03	$(1.0 \pm 0.0)\cdot 10^{-12}$	−69 ± 0	−69.05 ± 0.05

Table S8. Summary of parameters determined from ITC in water (25 °C). *N* is the molar ratio at the ITC record inflex point (interaction saturation point in the main text); Δ*H*º, Δ*G*º, −*T*Δ*S*º are defined per mole of hyaluronan basic unit.

Arginine Oligomer	Hyaluronan Molecular Weight (kDa)	*N* (-)	Δ*H*º (kJ/mol)	K_D (M)	Δ*G*º (kJ/mol)	−*T*Δ*S*º (kJ/mol)
Dimer	9	-	*3.10 ± 0.06	-	-	-
	1540	-	*3.894 ± 0.002	-	-	-
Tetramer	9	-	*3.44 ± 0.02	-	-	-
	1540	-	*4.13 ± 0.05	-	-	-
Octamer	9	-	*3.68 ± 0.06	-	-	-
	1540	-	*4.33 ± 0.04	-	-	-
Octamer with adjusted pH	9	-	*3.21 ± 0.17	-	-	-
	1540	1.5 ± 0.2	2.3 ± 0.4	$(1.5 \pm 1.3)\cdot 10^{-5}$	−28 ± 3	−31 ± 2
Dodecamer	9	1.02 ± 0.02	3.41 ± 0.05	$(4 \pm 2)\cdot 10^{-4}$	−19.4 ± 1.1	−22.8 ± 1.1
	109	0.91 ± 0.05	3.5 ± 0.2	$(2.9 \pm 1.1)\cdot 10^{-5}$	−26.1 ± 1.0	−29.5 ± 0.9
	137	0.88 ± 0.08	3.59 ± 0.19	$(3.9 \pm 0.9)\cdot 10^{-5}$	−25.3 ± 0.5	−28.9 ± 0.5
	680	0.89 ± 0.09	3.8 ± 0.2	$(3 \pm 2)\cdot 10^{-5}$	−25.8 ± 1.3	−29.7 ± 1.3
	1540	0.98 ± 0.02	4.33 ± 0.12	$(2.2 \pm 0.4)\cdot 10^{-5}$	−26.6 ± 0.5	−31.0 ± 0.6
Triacontamer	9	1.04 ± 0.04	2.16 ± 0.04	$(1.3 \pm 0.7)\cdot 10^{-5}$	−29 ± 2	−31 ±2
	16	1.11 ± 0.06	2.28 ± 0.04	$(3.8 \pm 1.7)\cdot 10^{-6}$	−31.2 ±1.1	−33.5 ±1.1
	109	1.09 ± 0.05	2.50 ± 0.14	$(5 \pm 3)\cdot 10^{-7}$	−36.2 ±1.6	−39 ±2
	137	1.11 ± 0.04	2.45 ± 0.11	$(4 \pm 7)\cdot 10^{-7}$	−38 ±5	−40 ±5
	310	1.03 ± 0.05	2.58 ± 0.05	$(4 \pm 3)\cdot 10^{-7}$	−38 ±5	−41 ±5
	680	0.97 ± 0.04	3.2 ± 0.1	$(3 \pm 5)\cdot 10^{-6}$	−39 ±8	−42 ±8
	1540	1.02 ± 0.11	3.5 ± 0.3	$(5 \pm 5)\cdot 10^{-7}$	−37 ±3	−40 ±3

*see text in part 3.2.

Table S9. Parameters determined for arginine decamer from ITC in water (25 °C). *N* is the molar ratio at the ITC record inflex point (interaction saturation point in the main text); ΔH^o, ΔG^o, $-T\Delta S^o$ are defined per mole of hyaluronan basic unit.

Binding Model	10 Arg·HCl				
	N (-)	ΔH^o (kJ/mol)	K_D (M)	ΔG^o (kJ/mol)	$-T\Delta S^o$ (kJ/mol)
9 kDa Hya one set of sites model	0.030 ± 0.002	15 ± 2	$(1 \pm 0)\cdot 10^{-12}$	−69 ± 0	−84± 2
9 kDa Hya first interaction event	1.12 ± 0.01	−0.29 ± 0.12	$(1.35 \pm 0.3)\cdot 10^{-5}$	−27.9 ± 0.6	−27.6± 0.4
9 kDa Hya second interaction event	0.31 ± 0.03	335 ± 0	$(6.0 \pm 2.8)\cdot 10^{-4}$	−21 ± 4	−353 ± 2
109 kDa Hya first interaction event	1.06 ± 0.01	22 ± 15	$(3.2 \pm 2.4)\cdot 10^{-4}$	−21 ± 3	−45 ± 11
109 kDa Hya second interaction event	0.30 ± 0.02	0.137 ± 0.006	$(6 \pm 1)\cdot 10^{-6}$	−56 ± 11	−56 ± 11
310 kDa Hya first interaction event	0.3 ± 0.03	16 ± 2	$(2.2 \pm 1.2)\cdot 10^{-4}$	−21 ± 2	−37 ± 0.4
310 kDa Hya second interaction event	1.10 ± 0.02	0.173 ± 0.03	$(3 \pm 3)\cdot 10^{-12}$	−67 ± 3	−66 ± 4
1540 kDa Hya first interaction event	0.4 ± 0.04	15 ± 3	$(5.7 \pm 1.4)\cdot 10^{-4}$	−18 ± 1	−35 ± 2
1540 kDa Hya second interaction event	1.09 ± 0.01	0.37 ± 0.11	$(3.2 \pm 1.2)\cdot 10^{-11}$	−65 ± 6	−65 ± 6

Table S10. Parameters for arginine dodecamer (hydrochloride form) titration in environments of different ionic strength determined from ITC (25 °C). *N* is the molar ratio at the ITC record inflex point (interaction saturation point in the main text); ΔH^o, ΔG^o, $-T\Delta S^o$ are defined per mole of hyaluronan basic unit.

Concentration of NaCl (mM)	680 kDa Hya + 12 Arg·HCl				
	N (-)	ΔH (kJ/mol)	K_D (M)	ΔG (kJ/mol)	$-T\Delta S$ (kJ/mol)
water	0.89 ± 0.09	3.8 ± 0.2	$(3 \pm 2)\cdot 10^{-5}$	−25.8 ± 1.3	−29.7 ± 1.3
10 mM	0.96 ± 0.05	1.5 ± 0.2	$(2.3 \pm 2.9)\cdot 10^{-6}$	−32 ± 4	−34 ± 4
50 mM	0.94 ± 0.04	1.4 ± 0.2	$(1.7 \pm 2.1)\cdot 10^{-5}$	−28.5 ± 2.8	−29 ± 3
100 mM	0.98 ± 0.14	0.62 ± 0.05	$(3 \pm 1)\cdot 10^{-5}$	−26.2 ± 1.5	−26.8 ± 1.5
150 mM	-	-	-	-	-

Table S11. Parameters for arginine decamer (hydrochloride form) titration in environments of different ionic strength determined from ITC (25 °C). *N* is the molar ratio at the ITC record inflex point (interaction saturation point in the main text); ΔH^o, ΔG^o, $-T\Delta S^o$ are defined per mole of hyaluronan basic unit.

Hyaluronan Molecular Weight / Concentration of NaCl	Hya + 10 Arg·HCl				
	N (-)	ΔH (kJ/mol)	K_D (M)	ΔG (kJ/mol)	$-T\Delta S$ (kJ/mol)
9 kDa / 10 mM	--	1.00 ± 0.11	--	--	--
1540 kDa / 10 mM	1.01 ± 0.10	0.52 ± 0.07	$(1.2 \pm 1.6)\cdot 10^{-5}$	−31 ± 7	−32 ± 7
9 kDa / 50 mM	--	0.283 ± 0.012	--	--	--
1540 kDa / 50 mM	0.95 ± 0.05	0.71 ± 0.06	$(4.5 \pm 2.2)\cdot 10^{-6}$	−31 ± 5	−31 ± 4

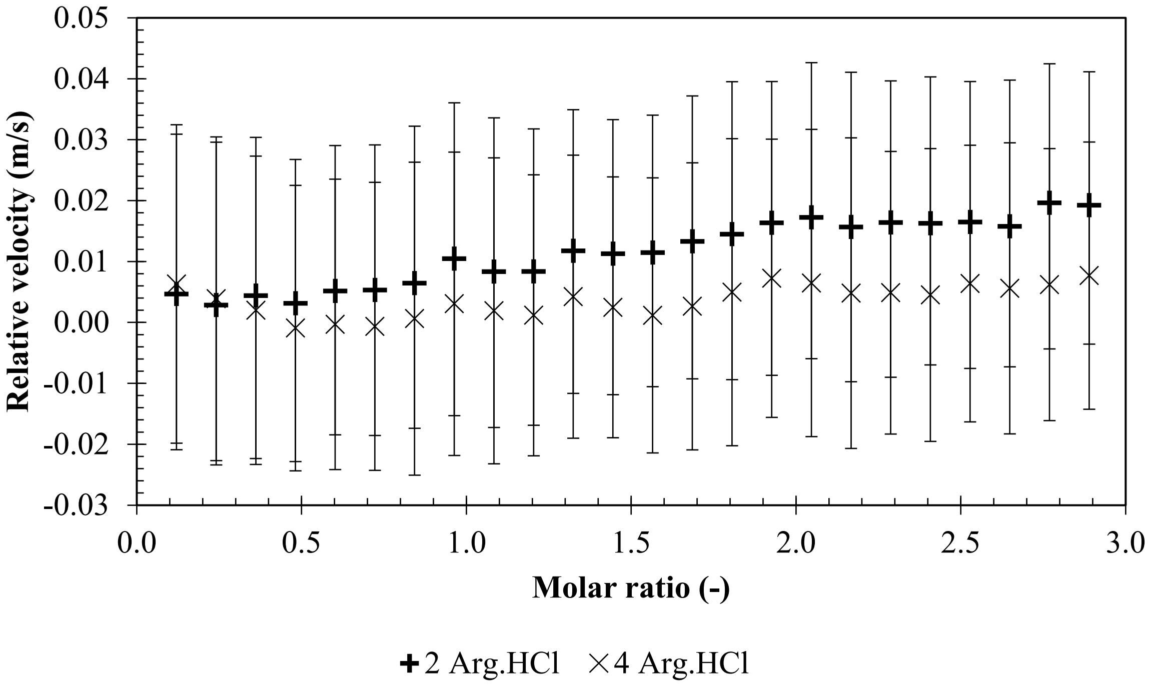

Figure S1. Relative ultrasonic velocity in dependence on molar ratio for titrations of arginine dimer and tetramer into hyaluronan of molecular weight 9 kDa in water. (11.6 MHz, 25 °C).

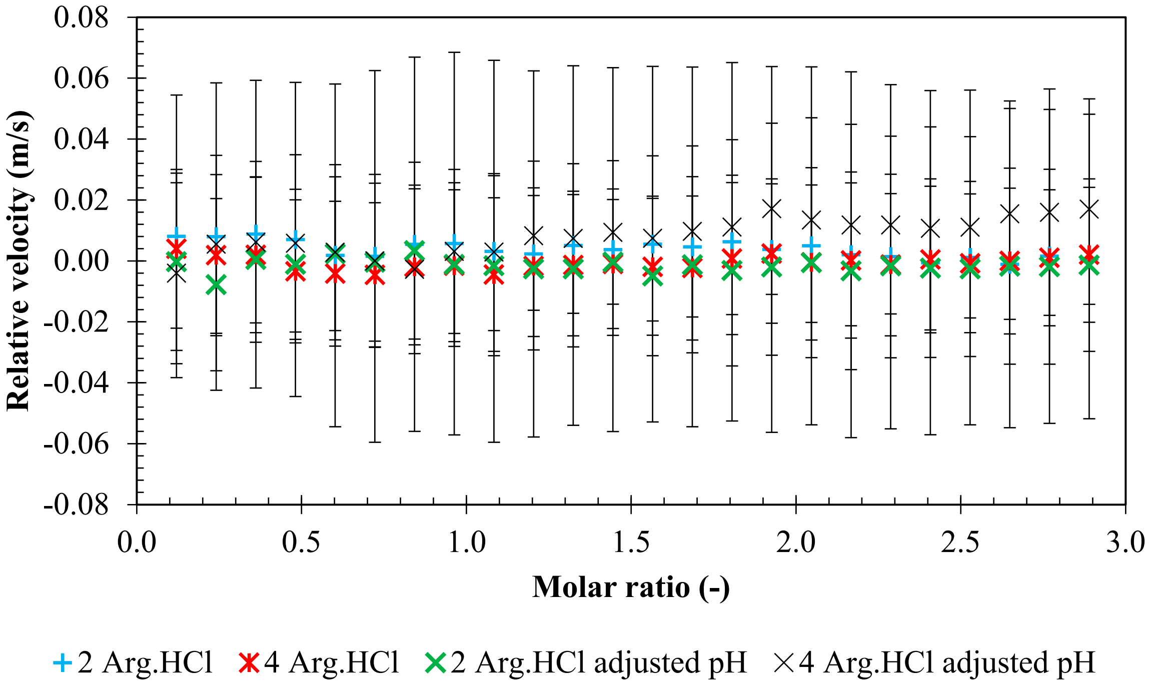

Figure S2. Relative ultrasonic velocity in dependence on molar ratio for titrations of arginine dimer and tetramer with and without pH adjustments into hyaluronan of molecular weight 1540 kDa in water. (11.6 MHz, 25 C).

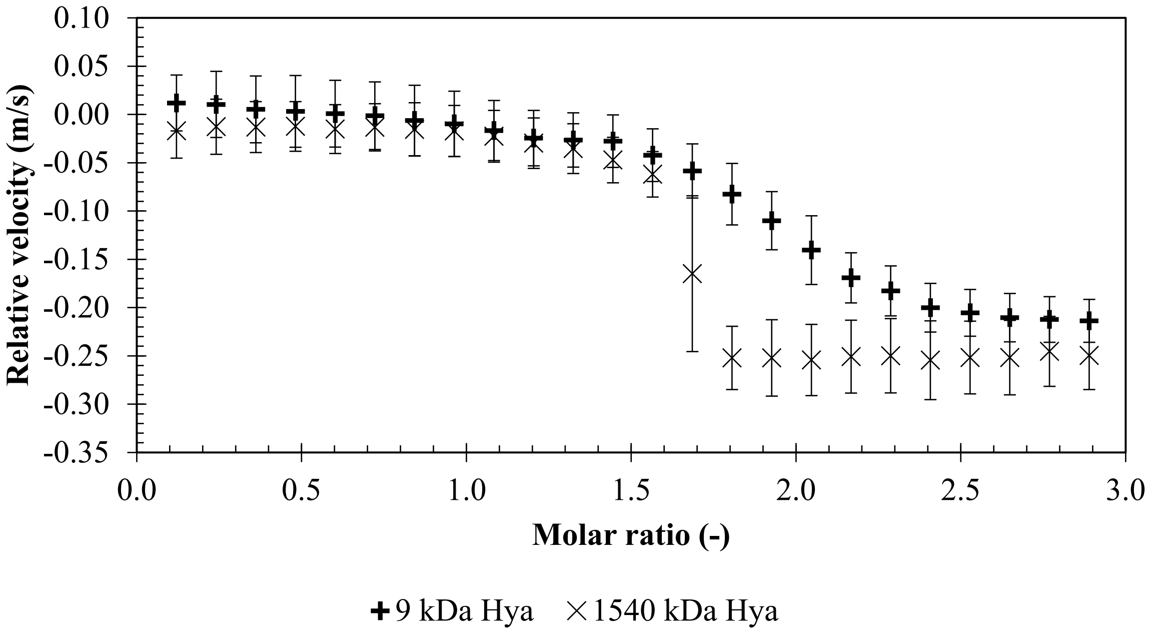

Figure S3. Relative ultrasonic velocity in dependence on molar ratio for titrations of arginine octamer with adjusted pH into hyaluronan of different molecular weights in water. (11.6 MHz, 25 °C).

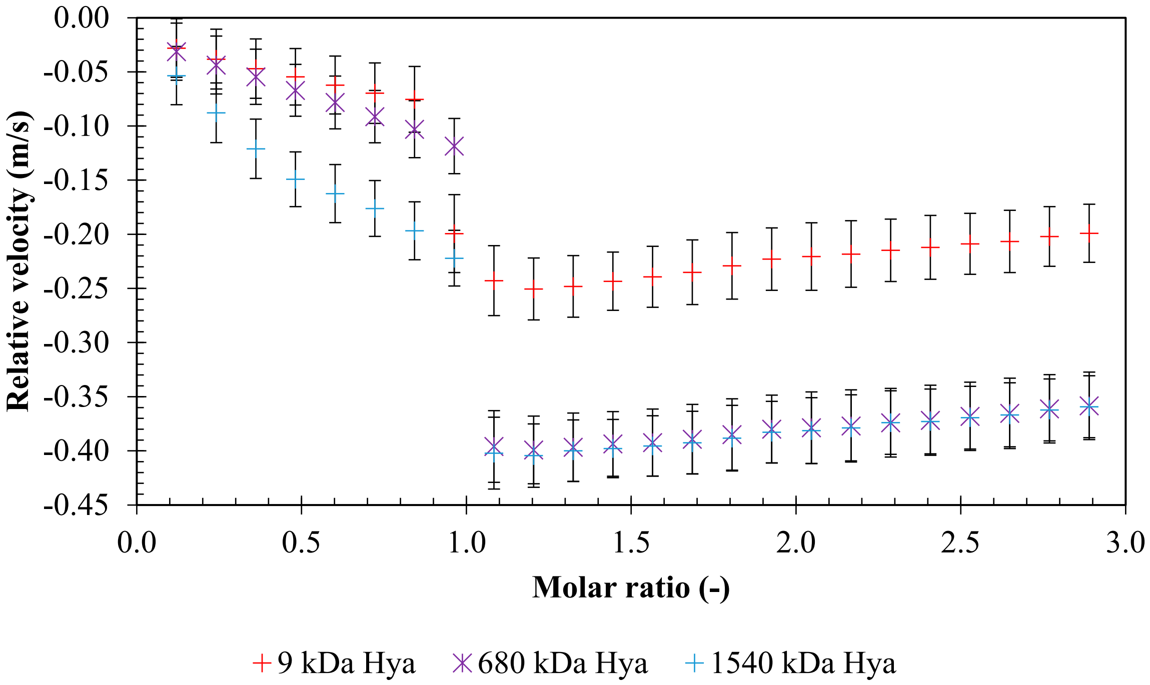

Figure S4. Relative ultrasonic velocity in dependence on molar ratio for titrations of arginine dodecamer into hyaluronan of different molecular weights in water. (11.6 MHz, 25 °C).

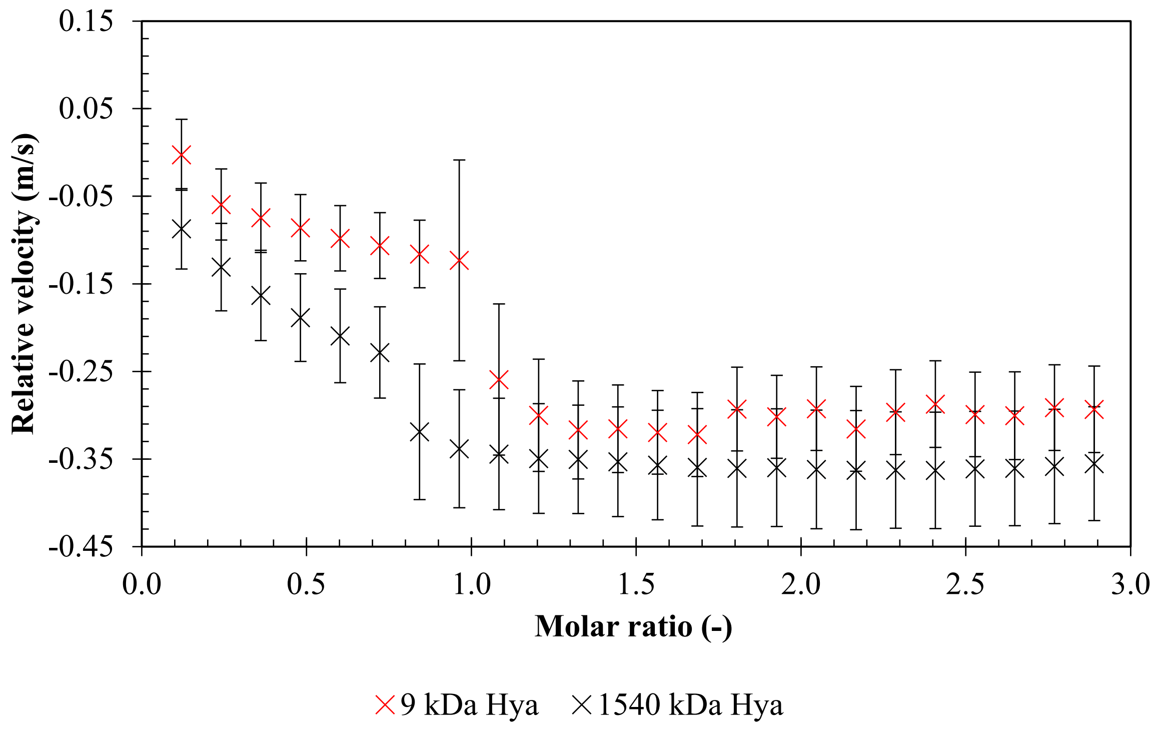

Figure S5. Relative ultrasonic velocity in dependence on molar ratio for titrations of arginine triacontamer into hyaluronan of different molecular weights in water. (11.6 MHz, 25 °C).

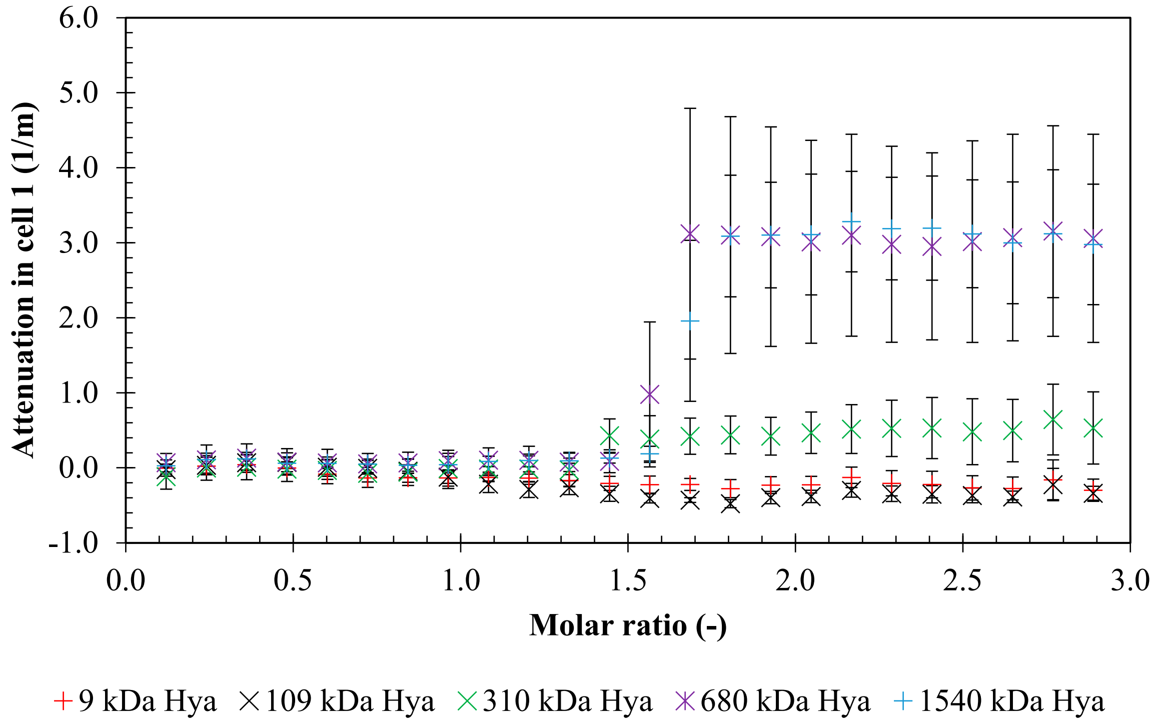

Figure S6. Attenuation in cell 1 in dependence on molar ratio for titrations of arginine triacontamer into hyaluronan of different molecular weights in PBS. (11.6 MHz, 25 °C).

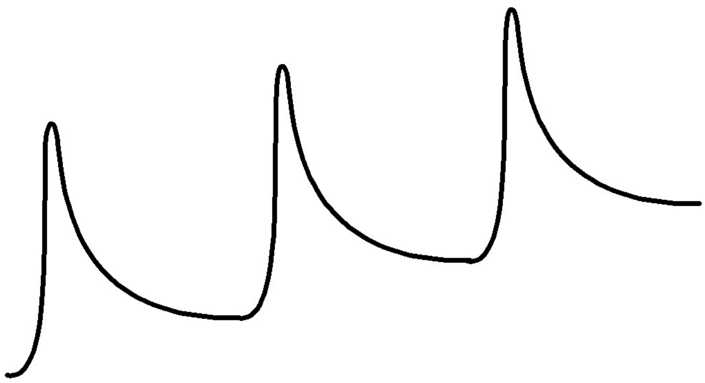

Figure S7. The stepped shift of the baseline due to a change in the thermal capacity of the system.

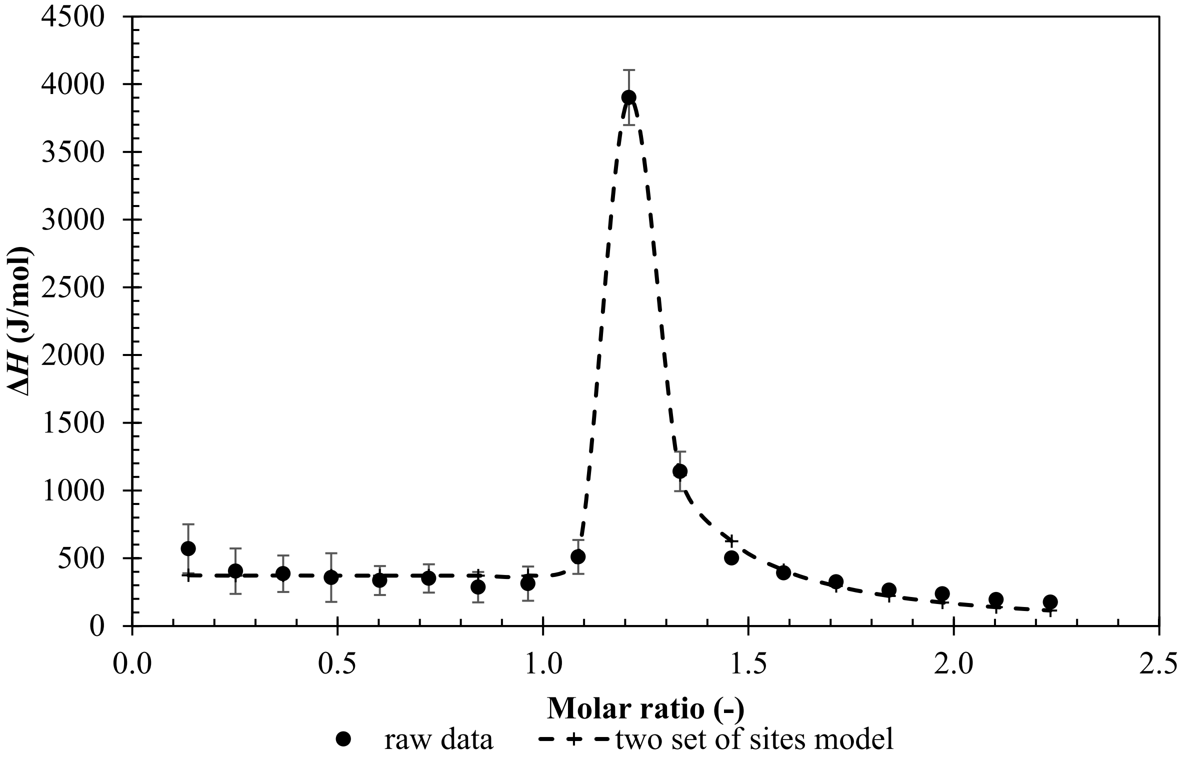

Figure S8. ITC records for the titration of arginine decamer in hydrochloride form into a 1540 kDa hyaluronan solution in water (25 °C). Two sets of site models were used for data evaluation.

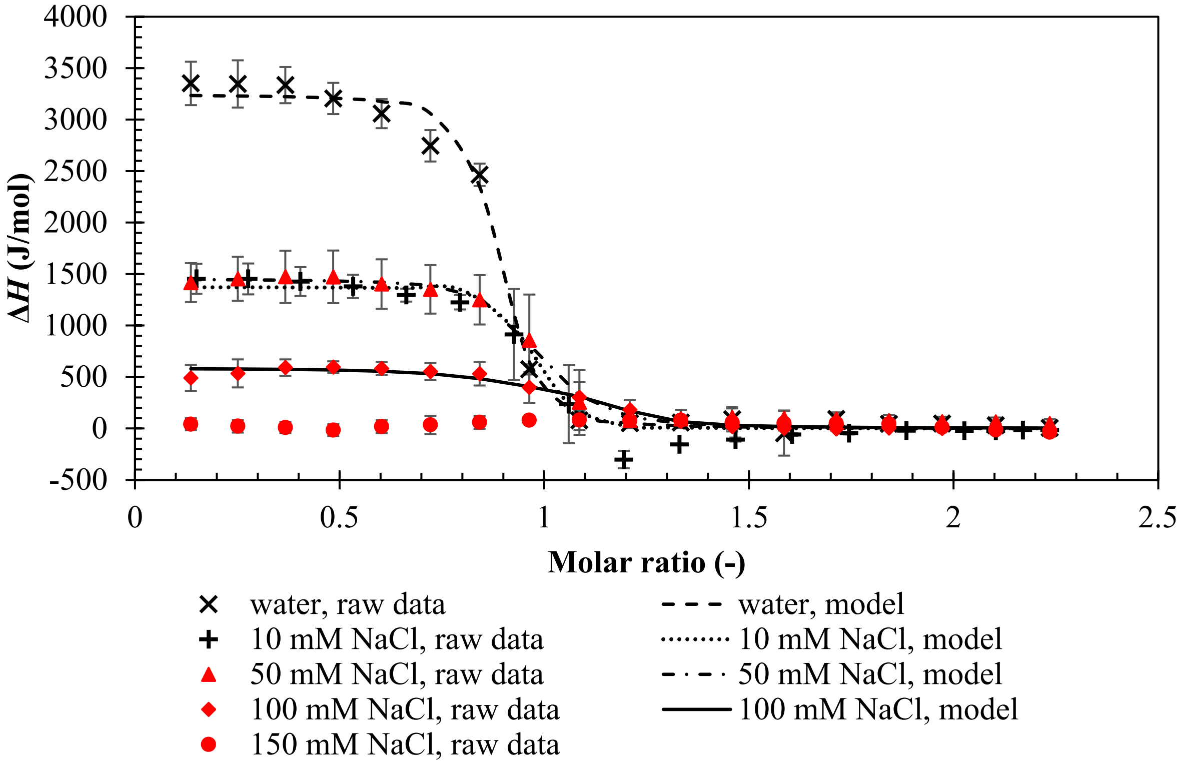

Figure S9. ITC records for the titration of arginine dodecamer in hydrochloride form into a 680 kDa hyaluronan solution in environments with different ionic strengths (25 °C). Single binding site models were used for data evaluation.

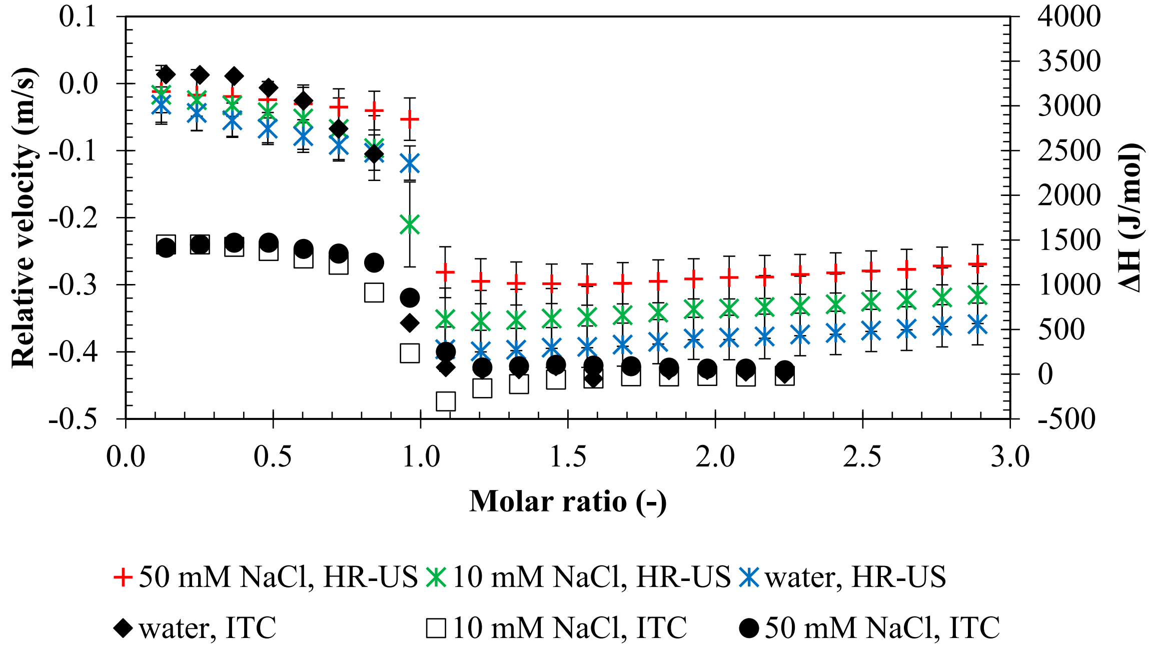

Figure S10. The comparison of HR-US and ITC titration records during the titration of arginine dodecamer in hydrochloride form into a 680 kDa hyaluronan solution in environments of different ionic strength. (11.6 MHz, 25 °C).

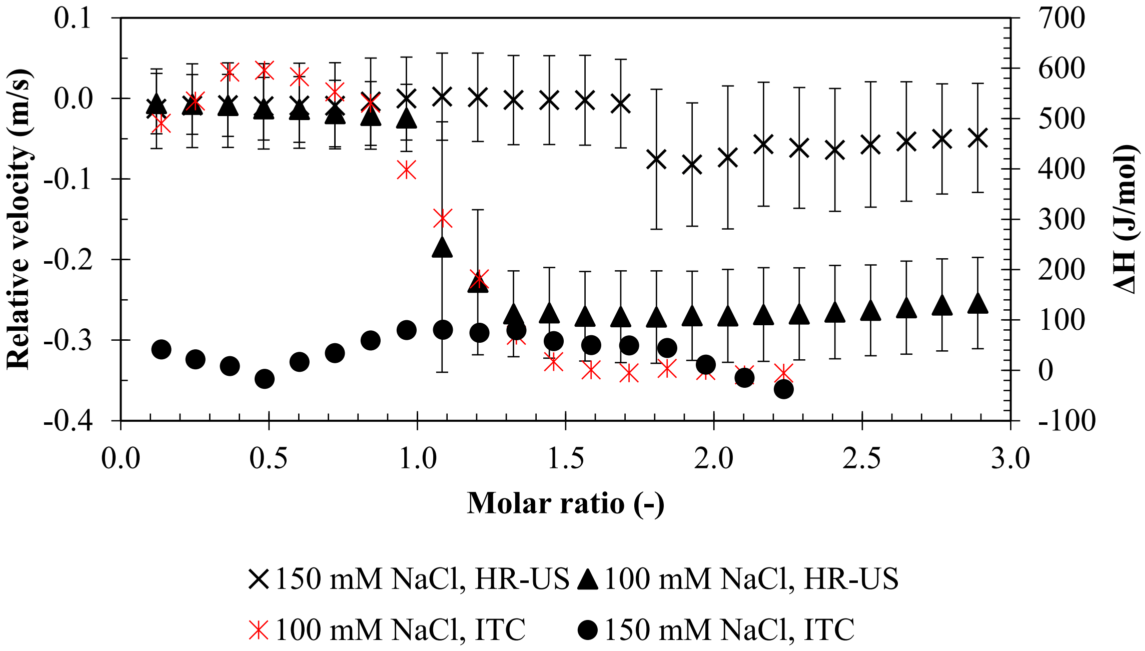

Figure S11. The comparison of HR-US and ITC titration records during the titration of arginine dodecamer in hydrochloride form into a 680 kDa hyaluronan solution in environments of different ionic strength. (11.6 MHz, 25 °C).

© 2020 by the authors. Licensee MDPI, Basel, Switzerland. This article is an open access article distributed under the terms and conditions of the Creative Commons Attribution (CC BY) license (http://creativecommons.org/licenses/by/4.0/).
